# Hypodermic Ergot in Post-Partum Hemorrhage

**Published:** 1875-01

**Authors:** P. C. Williams

**Affiliations:** Baltimore


Hypodermic Injection of Ergot—*By P. C. Williams, M.D., of Baltimore.*—* * * I have thus given three cases of post-partum hemorrhage which present three different conditions.

The first was a case of uncomplicated labor, but slow and tedious, producing great fatigue, which was the probable cause of the subsequent uterine relaxation and the hemorrhage. The administration of chloroform may also have been a predisposing cause of the hemorrhage, although I was careful, as I always am, to accompany the use of chloroform with the internal administration of ergot.

In the second case, chloroform was also administered, and its use was much prolonged by the necesity of tearing away the adherent placenta.

The prolonged use of chloroform and the adherent placenta were doubtless the causes of the flooding in this case, nor was it prevented by the free use of ergot by the stomach.

In the third case no chloroform was used, yet the flooding was quite as great as in either of the other cases.

In the first and third cases, various means were resorted to without the slightest effect, until ergot was given hypodermically. In the second case, nothing was used but the hypodermic injection of ergot.

In these three cases the effect of the hypodermic was almost instantaneous, and it was permanent.

It seems to me, therefore, that the hypodermic use of ergot offers a safe, prompt and efficient remedy for post-partum hemorrhage, and I earnestly advise its administration. In the cases here reported I used the fluid extract of ergot. It produced no abscesses, and was entirely satisfactory in its effects. According to the experiments of Dr. Squibb, of Brooklyn, ergotine does not represent the full therapeutic value of ergot, and therefore cannot be relied upon. Dr. Squibb, at the suggestion of Dr. Marion Sims, has made a *solid extract* which is very efficient, and has been frequently used in New York and elsewhere for the treatment of uterine fibroids. The solid extract is rendered fluid by "rubbing it up" with water, in the proportion of one grain of the extract to one minim of water, when it is available for hypodermic use.—*Medical and Surgical Journal.*

NOTE.—A half dram of the fluid extract of ergot was injected in the thigh in each case.—EDITOR.

